# Resilient entrepreneurs? — revisiting the relationship between the Big Five and self-employment

**DOI:** 10.1007/s11187-022-00686-7

**Published:** 2022-09-28

**Authors:** Petrik Runst, Jörg Thomä

**Affiliations:** Institute for Small Business Economics at the Georg-August-University Göttingen, Heinrich-Düker-Weg 6, 37073 Göttingen, Germany

**Keywords:** Entrepreneurship, Self-employment, Big Five, Personality, Prototypes, Profiles, D91, L26, M13

## Abstract

The Big Five personality traits and their influence on entrepreneurial action have been repeatedly studied using a trait-based approach. The present paper partly deviates from this perspective by analysing the role of personality prototypes in relation to entrepreneurship. This person-centred approach suggests that combinations of Big Five traits form individual personalities. By using data from the German Socio-Economic Panel (SOEP), we show that at least three prototypes can be identified, one of which — the resilient type — can be hypothesized to significantly increase the likelihood of entrepreneurial action. Our regression results provide evidence of a positive impact of this prototype on the likelihood of and transitioning into self-employment but not the likelihood of exit. We also show that the prototyping approach explains individual self-employment decisions over and above what can already be explained by the profiling approach, another person-centred Big Five approach. The paper concludes with implications for policy and research.

## Abstract (plain language summary)

Identifying entrepreneurial personalities — an improved person-centred approach to the Big Five personality traits advances our understanding of self-employment decisions. Previous studies emphasize a positive influence of the personality traits extraversion and openness on entrepreneurship. The present paper shows that the interaction of personality traits is also important. A resilient personality type that combines high values in the aforementioned traits with higher levels of conscientiousness, agreeableness and emotional stability has a positive impact on the likelihood to become self-employed. We also show that a resilient personality type explains self-employment decisions beyond what can already be explained by profiling, another person-centred Big Five approach. As a practical implication, advice from career or start-up consultants should not be based on profiling alone. Otherwise, too many entrepreneurs may be discouraged from their entrepreneurial endeavours. Generally speaking, self-employment decisions should incorporate personality aspects only as one among many relevant factors.

## Introduction

Based on the well-established literature on the broad Big Five personality traits (Digman, 1990; John et al., 1991, 2008; McCrae & Costa, 2008), a number of researchers have analysed the effects of such traits on entrepreneurship. Two basic approaches can be distinguished here. First, by using the *trait-oriented approach* (i.e. the Big Five traits are examined separately from each other), openness to experience or extraversion has been repeatedly found to exert a positive influence on the decision to start a business (Brandstätter, 2011; Shane et al., 2010; Zhao & Seibert, 2006), while agreeableness is found to increase the exit probability from self-employment (Caliendo et al., 2014). The relationship between the Big Five traits and more narrow traits — such as locus of control (LOC) or risk tolerance – has also been examined, showing that additional personality aspects besides the Big Five traits are relevant for predicting entrepreneurial decisions (Caliendo et al., 2014; Leutner et al., 2014).

Second, other entrepreneurship studies have taken a *person-oriented approach* to the Big Five Inventory. These are based on the observation that a combination of high levels of extraversion, conscientiousness, emotional stability, and openness and low levels of agreeableness is a good predictor of entrepreneurial activity. This particular configuration of Big Five traits has become known as the entrepreneurial personality profile (Schmitt-Rodermund, E. 2004; Obschonka et al., 2013; Obschonka & Stuetzer, 2017). In studies following this line of research, a hypothetical benchmark is generated that reflects the mentioned trait configuration. In a next step, the squared distance between an individual’s actual Big Five traits and this reference profile is calculated. According to Obschonka and Stuetzer (2017), the entrepreneurial personality profile is a robust predictor of self-employment decisions at both the individual and regional level. However, there is a debate about the practical implications of this profiling approach for the design of entrepreneurship education or business consulting. For example, Konon and Kritikos (2019) argue that while personality profiles based on hypothetical reference personalities may yield well-fitting regression lines, they are unsuccessful in making real predictions of future self-employment decisions, as the focus on a single profile cannot fully account for the stark heterogeneity of individuals who are prone to entrepreneurial activity.

In the last two decades, another person-oriented Big Five approach has emerged in the psychology literature, with findings that remain, with two exceptions (Caliendo et al., 2022a; Runst & Thomä 2022), unexploited by small business and entrepreneurship research. Instead of treating the Big Five traits as five independent motivators of human action, this approach posits that traits are synergistic with each other, in the sense that stable and empirically discernable interdependencies exist between the Big Five traits. Starting from this assumption, distinct types of individual personalities are measured, known as personality prototypes (e.g. Asendorpf et al., 2001; Boehm et al., 2002; Herzberg & Roth, 2006; Specht et al., 2014; Gerlach et al., 2018). By working with trait configurations within individuals instead of single traits, prototyping is somewhat related to profiling. However, the two empirical approaches start from opposite ends. Profiling uses a single combination of traits that has been empirically shown to be associated with entrepreneurial activity. Thus, profiling starts from the predictive end of the empirical process, which can be argued is like putting the cart before the horse. On the other hand, prototyping is based on frequently occurring configurations of traits in the overall population of individuals, not only entrepreneurs. Only after stable personality types — i.e. discernable combinations of traits that reflect the heterogeneous nature of individual personalities — have been identified will their effects on entrepreneurship or any other phenomenon be examined.

Profiling assumes that if a trait has been shown to exert an effect on entrepreneurial behaviour, the impact of this trait will be the same when combined with certain manifestations of other traits. However, this assumption may not always be correct. For example, Caliendo et al. (2014) find that the trait agreeableness does not affect entry or self-employment, whereas studies on the entrepreneurial personality profile assume a negative impact (Obschonka & Stuetzer, 2017; Obschonka et al., 2013). Nevertheless, the level of agreeableness may also positively affect entry and exit when it occurs in conjunction with higher levels of other traits. In fact, our empirical results suggest that higher agreeableness increases entry probabilities when combined with high values of extraversion but does not affect entry when co-occurring with low levels of extraversion.

The possibility of such conditional effects is easily overlooked when entrepreneurial personality profiles are based on average effect sizes of the five trait variables in a regression analysis instead of considering the variety of possible trait combinations. The existence of such conditional (or interaction) effects suggests that the mutual interplay of traits matters. However, instead of examining the myriad of all theoretically possible combinations between all Big Five traits, prototyping starts from the distinct configurations that actually exist in the general population with some frequency and regularity, i.e. personality prototypes. In other words, while the profiling approach — with its focus on one specific combination of traits — can be understood as a first important step towards measuring distinct types of entrepreneurial personalities, the prototype approach likely represents a further step in this direction as it takes account of the heterogeneous nature of entrepreneurship-prone personalities.

The contribution of this paper is twofold. First, we apply the prototype approach to studying the relationship between personality and self-employment decisions. In this way, our study complements the findings of Caliendo et al. (2022a) about the effects of personality prototypes on hiring decisions of early-stage entrepreneurs and the results of Runst and Thomä (2022) on the self-selection of small business owners into different modes of firm-level innovation, contingent on the Big Five personality prototype. To test and demonstrate the validity of our approach, we empirically derive personality prototypes from a large longitudinal dataset using latent profile analysis and a cluster analysis. Second, we investigate whether the prototype approach can expand upon the explanatory power of the profiling method. Our results have relevant practical implications in the context of entrepreneurship education and business consulting, as they suggest that the use of personality profiles should be combined with a focus on specific entrepreneurial personality prototypes to increase the effectiveness of measures and services. In doing so, we aim to respond to Konon and Kritikos (2019), who identify a need for research regarding ‘what kind of metric methods should be used that take the heterogeneity among individuals better into account [than the profiling approach]’ (p. 14).

## Conceptual background

### Big Five personality traits and entrepreneurship

The Big Five Inventory (John et al., 1991, 2008) represents the most widely used measure of personality traits, and it has been employed extensively in the field of personality psychology and beyond. It contains the following five elements. The trait *extraversion* measures the extent to which an individual enjoys social interaction and possesses the corresponding social skills. Extraverted individuals are outgoing and communicate frequently. An individual with a high level of *agreeableness* tends to shy away from conflicts and has a more forgiving attitude towards others. Such an individual prefers cooperation to competition in social relationships and he or she is careful in his/her choice of words to avoid affronting others. A highly conscientious person is diligent in his or her tasks and has a higher achievement orientation. Due to their high level of *conscientiousness*, such persons are always planning ahead, prefer efficiency and pay close attention to details. The trait of *emotional stability* (opposite: neuroticism) is related to having fewer mood swings, less anxiety and fewer instances of feeling sad, hopeless or guilty. An individual with high levels of emotional stability is also more resilient in the face of setbacks and less vulnerable to psychological stressors. Finally, a person who is open to experience displays interest in novelty, variety and creativity. Higher levels of *openness* are associated with not liking routines and repetitive tasks, as well as higher degrees of active imagination.

It is theoretically plausible to draw a connection between these five traits and entrepreneurial action (e.g. Brandstätter, 2011; Caliendo et al, 2014; Zhao et al., 2010). For example, starting a business involves new ways of doing things, serving a market that either has not existed before or satisfying demand in a better way than before. Individuals who are open to experience are more likely to recognize such business opportunities as well as acting upon them, as entrepreneurs with creativity and a willingness to propel innovative changes. Similarly, entrepreneurial action is highly social in nature (Sarasvathy, 2001; 2009) and should therefore be more appealing to extraverted individuals who are more likely to communicate, create and maintain social connections with different types of external stakeholders necessary for business formation and success. While the preference for routine and repetition actions in conscientious individuals could easily reduce the likelihood of starting a business, conscientious business owners’ attention of to detail, strong work motivation and efficiency should certainly increase entrepreneurial performance at the growth stage of new ventures. A higher level of emotional stability can be advantageous in terms of resilience when the entrepreneur confronts challenges, stressful situations or obstacles that need to be overcome in the process of setting up and maintaining a business. Finally, agreeableness could be expected to be negatively related to entrepreneurship, as agreeable individuals are often less inclined to be sufficiently strong willed in the face of opposing viewpoints and arguments, acquiescing too quickly, which thereby undermines creative change processes in the context of entrepreneurship. On the other hand, as entrepreneurship is a social process (Sarasvathy, 2001; 2009), a low value of agreeableness can exacerbate social conflict and deter potential partners from cooperating with the prospective entrepreneur.

A number of empirical studies have followed a trait-oriented approach to examine the relationship between the Big Five and entrepreneurial action. Accordingly, they have established robust links between single traits and entrepreneurship. Higher levels of extraversion and openness and — to a lesser extent — emotional stability and conscientiousness are reliable predictors of entrepreneurial intention and performance (e.g. Ciavarella et al., 2004; Zhao & Seibert, 2006; Zhao et al., 2010; Brandstätter, 2011; Caliendo et al., 2014). Some evidence suggests that higher levels of agreeableness increase the likelihood of exit (Caliendo et al., 2014). Some entrepreneurship studies have also related narrow traits such as achievement orientation, locus of control and risk tolerance to the Big Five traits, showing that both broad and narrow traits have explanatory power for predicting entrepreneurial decisions (Caliendo et al., 2014; Leutner et al., 2014).

Finally, by taking a person-oriented approach to the Big Five, the profiling literature has repeatedly found that the specific combination of high levels of extraversion, openness, conscientiousness and emotional stability and low levels of agreeableness — i.e. the entrepreneurial personality profile — is positively associated with the decision to enter self-employment (Obschonka & Stuetzer, 2017; Obschonka et al., 2013; Schmitt-Rodermund, 2004).

### The prototype approach

As has been noted, previous entrepreneurship research on person-oriented investigations into the Big Five has focused on the profiling approach. However, in the last two decades, a second body of literature concerning person-oriented Big Five analyses has unfolded within the field of psychology (Asendorpf et al., 2001; Boehm et al., 2002; Fruyt et al., 2002; Schnabel et al., 2002; Herzberg & Roth, 2006; Meeus et al., 2011; Specht et al., 2014; Gerlach et al., 2018), which holds relevant implications for entrepreneurship and small business research but — apart from Runst and Thomä (2021) — remains largely untapped. This literature does not deal with separate individual traits but rather examines the statistical clustering or co-occurrence of traits in the general population of individuals. In other words, are there certain combinations of Big Five traits — labelled as prototypes — that are more likely to manifest themselves within the personality of individuals? The number of identified prototypes varies between three (Asendorpf et al., 2001; Meeus et al., 2011), four (Specht, 2014, Gerlach et al., 2018), and five (Kerber et al., 2021) whereas the empirical evidence tends towards the first number.

However, regardless of which solution was found, a particular personality type — labelled as the ‘resilient type’ (Asendorpf et al, 2001), also called the ‘role model’ (Gerlach et al., 2018) — has been clearly identified in all of these studies (for a literature review, see Kerber et al., 2021). This prototype is characterized by high values in all Big Five traits. According to Asendorpf et al., (2001, p. 175), the resilient type refers to a person’s ability ‘to respond flexibly, rather than rigidly to changing situational demands, particularly stressful situations.’ In three-type solutions, the other two prototypes that have been identified are ‘over-controllers’ (i.e. high values of conscientiousness but lower values of openness and extraversion) and ‘under-controllers’ (i.e. low values in all traits, including emotional stability). Four- and five-type solutions differ from three-type solutions in terms of the identification of under- and over-controllers or certain subgroups thereof (Gerlach et al., 2018). In this context, the degree of self-control refers to an individual’s ‘tendency to contain versus express emotional and motivational impulses (strong control vs. weak control)’ (Asendorpf et al., 2001, p. 175).

The prototype approach is inherently based on the idea that there are certain synergies between separate Big Five traits. For example, Runst and Thomä (2021) provide empirical evidence that small business owners’ personality traits complement each other in the context of firm-level innovation. According to their results, a small firm is more likely to successfully implement an informal mode of innovation, which places a special emphasis on interactive learning and cooperative relationships when the owner is of the resilient type. Such synergies can also be expected in the context of entrepreneurship. For example, while extraversion and openness have widely been found to positively affect the probability of entry into self-employment, in terms of new venture performance, the founder’s degrees of conscientiousness and emotional stability should play a complementary role as high degrees of achievement motivation and a pronounced ability to cope with stress should also be important for the success of entrepreneurs (Zhao et al., 2010).

Perhaps the best example of such synergies is the ambiguous role of an entrepreneur’s degree of agreeableness. As noted above, in the profiling literature, a negative role is assigned to the trait of agreeableness in terms of entrepreneurial action. This perspective speaks to a conception of the entrepreneur as the lone maverick that pursues his/her vision of innovation and changes quite ruthlessly and overcomes obstacles in the form of resisting voices by not deferring to others in the face of conflict. Interestingly, this caricature of a visionary dynamic change agent is at odds with what qualitative research tells us that the process of entrepreneurship actually looks like (Sarasvathy, 2001, 2008). In fact, the entrepreneurial process has been described as a social one, embedded within and reliant upon a viable network of customers, suppliers etc. Instead of the lone maverick, Sarasvathy (2001, 2008) metaphorically describes the entrepreneur as a quilt maker, stitching various stakeholders and their ideas together into a joint fabrication of opportunity, generating a community of co-conspirators in the process. Such a conception of the entrepreneur would not suffer from high levels of agreeableness. In fact, such a personality trait would benefit the entrepreneurial process, as the other members of the emerging new venture’s network would be more willing to engage and trust an agreeable entrepreneur given that he/she would be more likely to incorporate their various views and interests. Hence, agreeableness may exert different effects on entrepreneurship, depending on the context and the interplay with other Big Five traits involved. Indeed, the ability of the prototype approach to consider these heterogeneities among individuals and condense them into certain dominant personality types reflects precisely its strength.

### The resilient type and entrepreneurial action

As already mentioned, one trait configuration that has been consistently identified in the prototype literature is the resilient type. It refers to individuals who are ‘able to resourcefully adapt to changing situations and circumstances, to tend to show a diverse repertoire of behavioral reactions and to be able to have a good and objective representation of the “goodness of fit” of their behavior to the situations/people they encounter. This good adjustment may result in high levels of self-confidence and a higher possibility to experience positive affect’ (Kerber et al, 2021, p. 3). Such a personality type can be expected to be likely to engage in entrepreneurial action. For example, Runst and Thomä (2021) show that small firms with owners whose personality resembles the resilient type are more likely to successfully implement a non-R&D-based mode of innovation. Hence, we hypothesize that individuals of the resilient type are more likely to enter and remain self-employed, as they will create and maintain the necessary social ties (extraversion, agreeableness), diligently plan and execute required actions (conscientiousness), remain calm in the face of adversity (emotional stability) and display an open attitude toward novelty and change (openness).

On the other hand, over-controllers have been described as constrained and inhibited in their behaviour, limited in their emotional expressivity and overly cautious in decision-making (Kerber et al., 2021). The social nature of entrepreneurship should render it less likely for such an individual to enter into self-employment.^1^ In addition, entrepreneurial action requires the capacity to make decisions under stressful and uncertain situations, meaning that a certain degree of emotional stability is needed for entrepreneurs to succeed (Zhao et al., 2010). On the other hand, under-controllers display high time discounting and are therefore often unable to delay gratification to receive larger gains in the future. Moreover, they tend to be ‘relatively unattached to social standards or customs’ (Kerber et al., 2021, p. 2). For example, the inability to delay gratification has been connected to various negative economic or social outcomes (see DellaVigna, 2009), such as lower scores on standardized tests (Mischel et al., 1989), lower educational attainment (Ayduk et al., 2000), higher body mass indexes (Schlam et al., 2013) and lower savings (Ashraf et al., 2006). In terms of social interactions, the results of Runst and Thomä (2021) imply that small business owners of the under-controlled personality type have a low likelihood of implementing a mode of learning and innovation at the firm level that builds on interactive learning and cooperation with external partners. In a similar manner, we expect under-controlled individuals to be less inclined to entrepreneurial action.

## Data and methods

### German SOEP

We use data from the Socio-Economic Panel (SOEP) for 2005 to 2019.^2^ The SOEP is a large and representative annual longitudinal household survey among individuals throughout Germany that has been used in entrepreneurship research on the Big Five (e.g. Caliendo et al., 2014) as well as psychology research on Big Five personality traits (e.g. Specht et al., 2014).^3^ The SOEP contains repeated questions on work, health, and well-being as well as additional non-repeated modules. Starting in 2005, the survey also includes a fifteen-item Big Five Inventory (BFI) at regular intervals (i.e. the five survey years 2005, 2009, 2013, 2017, and 2019). It has been shown that small item scales such as the BFI-15 retain significant levels of reliability and validity compared with longer versions such as the BFI-44 (Rammstedt & John, 2007). About 11,700 individuals fully answered all personality questions on the Big Five traits in 2005. As the survey has increased in sample size since then, there are about 14,900 complete Big Five observations in 2019. The dataset also provides information on the survey respondents, such as age, citizenship, educational and vocational degrees. This enables us to use information on self-employment status and transition as a measure for entrepreneurial action. There are ten LOC items in the SOEP (for the years 2005, 2010, and 2015). A factor analysis confirms that they can be reduced to one single factor. A positive loading on the LOC factor corresponds to an internal LOC orientation (i.e. the person believes in their own self-efficacy), and a negative loading relates to an external LOC (i.e. the person believes their life remains largely unaffected by his or her choices). The corresponding factor score — which we use in our regressions — represents an optimally weighted linear combination of these values.

Following Caliendo et al. (2014), the following analysis is limited to individuals between the ages of 19 and 59 to ‘to avoid possible confounding effects due to early retirement decisions’ (ibid, p. 795). Moreover, invalidity pensioners, students (including vocational education and training), farmers, family workers, civil servants and military members are removed from the sample. Apart from that, we do not include observations from the SOEP ‘Refugee Samples’ 2016 and 2017 in our analysis to ensure sample consistency over time, and because there are marked personality differences between the specific group of refugees and the general population in Germany (Brücker et al., 2016), which could otherwise distort the prototyping results.

### Methods

#### Overview

Our empirical analysis proceeds in three main steps. First, entrepreneurial profiles are derived from the SOEP survey data based on the individual manifestations of the Big Five survey items (see Sect. 3.2.2). Second, we generate personality prototypes. To ensure that the results of the prototype identification are valid, we derive them in two different ways, first via a latent profile analysis (LPA, see Sect. 3.2.3) and additionally — for the purpose of robustness testing — by applying a cluster analysis (see Sect. 3.2.4). On this basis, a longitudinal data set is created for the 2005–2019 period by replacing missing values in years without the Big Five module with values from the last available year.^4^

The five personality dimensions are extracted from fifteen survey items by generating factor scores for each trait. As an example, factor loadings for 2005 are presented in the Appendix (Table 5), and they conform to well-known patterns (e.g. Hahn et al., 2012; Lang et al., 2011).^5^ The factor scores are used in the LPA/cluster analysis as metric Big Five variables to generate the personality prototypes. Third, the profile and prototype variables both serve as variables in a regression analysis (see Sect. 3.2.5) on the determinants of different entrepreneurial actions (i.e. the self-employment status, the probability of entry/exit and the number of entries).

#### Entrepreneurial profile

We follow Obschonka et al. (2013) and Obschonka and Stuetzer (2017) by defining an entrepreneurial reference profile of the highest possible values on the traits’ original scales of extraversion, conscientiousness, emotional stability and openness and the lowest possible value on the agreeableness scale (${Y}_{k}$). Their reference profile is derived from the empirically established links between the single Big Five traits and entrepreneurship activity. In other words, when regressing self-employment decisions on the Big Five traits, a positive and significant coefficient leads to a high reference value, and correspondingly, a negative coefficient leads to a low reference value. We then calculate each individual’s semblance to the entrepreneurial profile by summing up the squared distances between the actual trait value (${X}_{itk}$) and its corresponding reference value (${Y}_{k}$), where index *k* refers to trait one to five.

$$\mathrm{Entrepreneurial\;Profile}= \sum_{k=1}^{5}{{(X}_{itk}-{Y}_{k})}^{2}$$

#### Prototyping: LPA

**Fig. 1:**
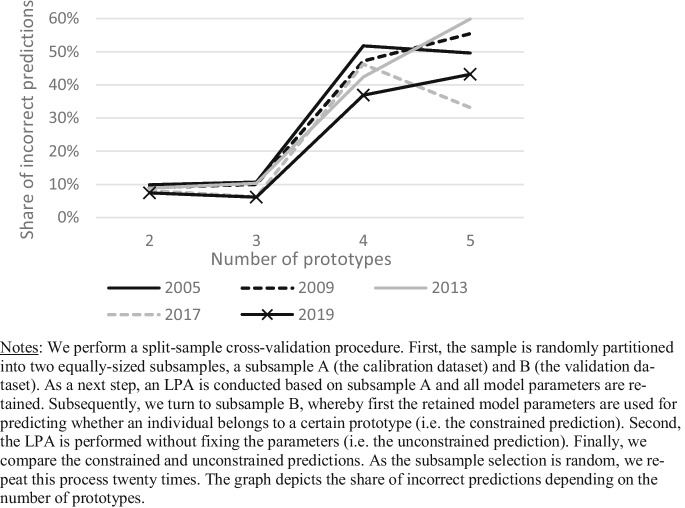
Distance to the entrepreneurial profile, by prototype

We follow Specht et al. (2014),  Asendorpf et al. (2001), and Asendorpf (2015) in performing a latent profile analysis (LPA) based on the derived factor scores on the Big Five traits, separately for each year in 2005, 2009, 2013, 2017 and 2019. As Specht et al. (2014) state, the aim of this typological approach ‘is to identify a preferably parsimonious number of personality types that allow for broad categorizations of individuals’ (p. 5). To determine the number of prototypes (*k*) in the model, we first run multiple LPAs, using two to five types. The Akaike information criterion (AIC) and Schwarz’s Bayesian information criterion (BIC) provide a statistic that can be used to assess the model’s fit, with lower values indicating a better fit. However, as is typical with these criteria, the AIC and BIC continuously decline when the number of prototypes *k* in the model rises. Masyn (2013) states in this regard that ‘because none of the information criteria are guaranteed to arrive at a single lowest value corresponding to a k -class model with k < k_max, these indices may have their smallest value at the k_max-class model’ (p. 572). We therefore perform a split-sample cross-validation procedure. First, the sample is randomly partitioned into two equally sized subsamples, subsample A (the calibration dataset) and B (the validation dataset). As a next step, an LPA is conducted based on subsample A, and all model parameters are retained. Subsequently, we turn to subsample B, whereby first, the retained model parameters are used for predicting whether an individual belongs to a certain prototype (i.e. the constrained prediction). Second, the LPA is performed without fixing the parameters (i.e. the unconstrained prediction). Finally, we compare the constrained and unconstrained predictions. As Masyn (2013) writes, ‘if the parameter estimates obtained from the k -class model fit to subsample A, then provide an acceptable fit when used as fixed parameter values for a k-class model applied to subsample B, then the model validates well and the selection of the k-class model is supported’ (p. 572–573). As the subsample selection is random, we repeat this process twenty times, separately for each survey year that contains BF items (2005, 2009, 2013, 2017 and 2019). The average share of incorrect predictions remains identical when moving from two to three prototypes (8.7% see Fig. 1). Thus, the three-type solution yields more descriptive variety without losing predictive accuracy. When moving from a three- to a four-prototype model however, we observe a sharp increase in the average share of incorrect predictions across all years (8.7 to 44.9%). Moving from four to five types further lowers predictive accuracy (48.3% incorrect predictions). We therefore conclude that a model containing three prototypes fits the data best. Each individual in the sample is assigned to one prototype only, based on its highest-class probability.

#### Robustness test: cluster analysis

In addition to LPA, clustering methods have also been used in previous research to identify personality prototypes (see e.g. Herzberg & Roth, 2006; Specht et al, 2014; Runst & Thomä, 2021). We resort to cluster analysis as it is well suited to check the robustness of the LPA results. Following Herzberg and Roth (2006), our clustering procedure comprises two steps. First, Ward’s hierarchical clustering is used to decide on the number of clusters to be formed. In this method, ‘the distance between two clusters is the sum of squares between the two clusters summed over all variables’ (Hair et al., 1998, p. 496). On this basis, the increase in within-cluster sum of squares is minimized over the stages of the clustering procedure. To determine the optimal number of clusters, we employ dendrograms showing the hierarchical relationship between the individual’s manifestations of the Big Five factors scores and apply two common cluster-stopping rules (Calinski/Harabasz pseudo-F index and Duda-Hart index). Across all survey years, as in the case of the LPA, the results speak in favour of a three-cluster-solution, although it should be noted that in some years, a four-cluster solution would have also been possible. However, in order to ensure consistency and comparability over the years regarding the LPA results, and because the particularly relevant group of the ‘resilient type’ clearly emerges in both solutions, we opted for three-prototype clusters. As a second step, we then conduct a k-means cluster analysis for each of the relevant survey years, where the cluster centroids of the Ward solution serve as initial seed points of the non-hierarchical clustering procedure. In this way, the benefits of hierarchical clustering in determining the number of clusters are combined with the advantages of non-hierarchical cluster analysis in fine-tuning ‘the results by allowing the switching of cluster membership’ (Hair et al., 1998, p. 498).

#### Regression analysis

We follow Caliendo et al., (2010, 2014) by estimating a logit model of the transitional probability of entry and exit conditional on the length of the pre-transition state. For the dependent variable ‘entry’, we therefore include the length of the employment or unemployment spell and drop all other individuals who are not employed or unemployed. For the dependent variable ‘exit’, we include the length of the self-employment spell, dropping all individuals who are not self-employed. As specified by Caliendo et al. (2014), spell length also enters the equation in quadratic and cubed form to capture non-linear effects. In the entry equation, we also interact spell duration with the pre-entrepreneurship state (employment or unemployment). We run normal logit models for the dependent variable ‘self-employed’. The dependent variable ‘Number of entries’ functions in the sense of a count variable, and we use OLS estimations in this case. Within our panel dataset, this variable gradually increases by one each time a person enters or re-enters the status of self-employment, and therefore captures serial entrepreneurship. In Number of entries specifications, we also add a control variable which records the number of years an individual has already been observed in the panel at time *t*, as the length of the observation period will directly affect the number of total entries of a person at time *t*. Depending on the model specification, we use entrepreneurial profiles, prototypes and a number of determinants known from the entrepreneurship literature as independent variables (see Table 7 in the Appendix).

## Empirical results

### Bidirectional interaction effects

**Fig. 2:**
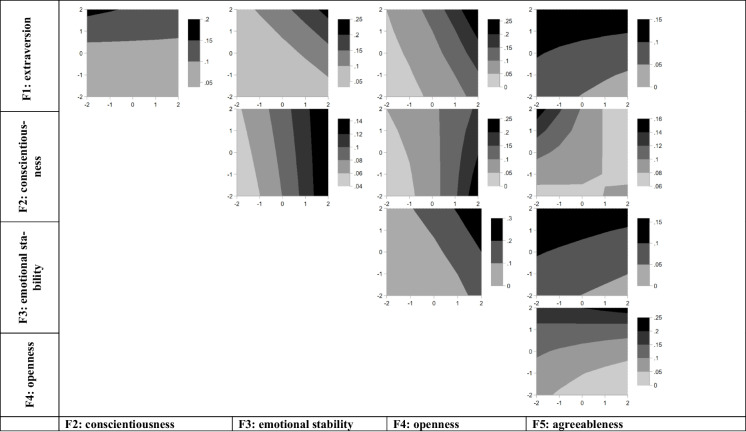
Interaction effects after logit regression, predicted probabilities (Dep. var. self-employed)

**Fig. 3:**
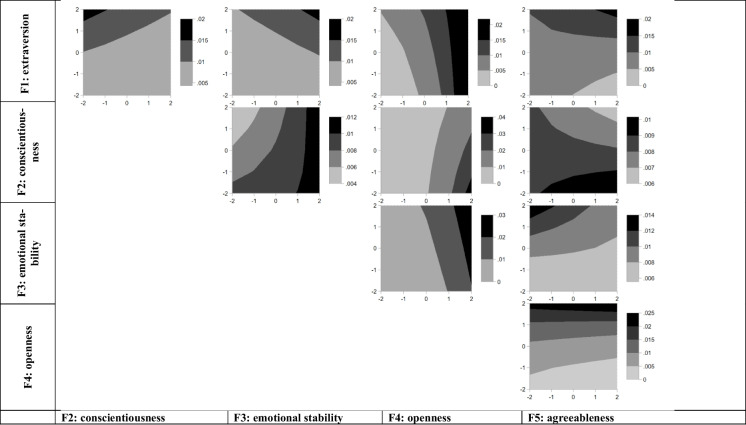
Interaction effects after logit regression, predicted probabilities (Dep. var. entry)

**Fig. 4:**
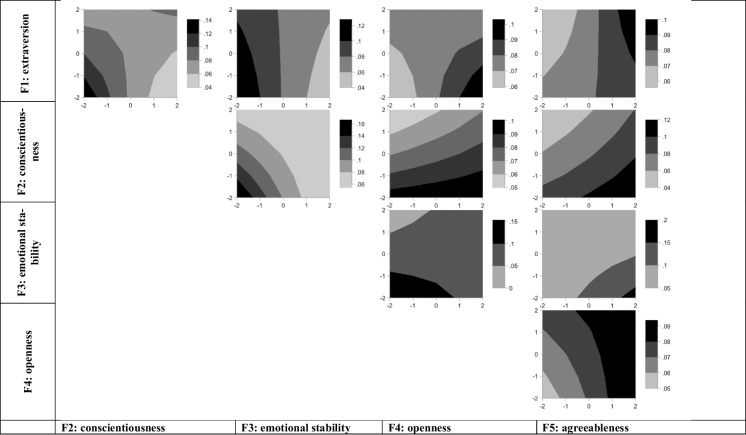
Interaction effects after logit regression, predicted probabilities (Dep. var. exit)

We demonstrate the importance of considering the mutual interplay between different personality traits in addition to single traits’ manifestations by regressing the dependent variables self-employment, entry and exit against all possible two-way interactions of the Big Five traits.^6^ The average predicted probabilities resulting from each pair of Big Five traits are shown by using contour plots (see Figs. 2, 3 and 4 in the Appendix). An illustrative example is the combined effect of agreeableness and emotional stability on self-employment (Fig. 2). If emotional stability is medium, a change in agreeableness has no influence on the probability of self-employment. Higher agreeableness only has a negative effect on the probability of self-employment if emotional stability is low. Similarly, at average levels of conscientiousness, there is no relationship between agreeableness and the probability of entry into self-employment (Fig. 3). However, agreeableness seems to increase the probability of entry when conscientiousness is low, and it reduces the probability of entry when conscientiousness is high. These findings speak for the existence of interaction effects between different Big Five traits in terms of self-employment decisions. Since the number of potential interaction effects would considerably increase if three-, four- and five-way interactions were included, in the following, we identify those combinations of Big Five traits that occur particularly frequently in individuals in the general population (i.e. personality prototypes).

### Prototypes: resilients, over- and under-controllers.

#### Results from the LPA

As stated above, a three-prototype solution fits the data best, which is in line with the majority of psychological research on the prototype approach. Table 1 lists the mean values for each Big Five personality trait by prototype. The corresponding characteristics are consistent with previous findings (see Sect. 2.2). Under-controllers are marked by low values in all Big Five traits, especially extraversion, conscientiousness, and agreeableness. Individuals of the over-controlled personality type are characterized by above-average values of conscientiousness, and agreeableness, while the remaining traits are close to the sample mean. Finally, the resilient type displays high values for the traits extraversion, conscientiousness, agreeableness and openness and above-average values for emotional stability. In all cases, the trait means of the resilient type are higher than those of the other two personality types. This overall pattern is consistent across all years and fits well with the study of Caliendo et al. (2022a), which also identifies prototypes based on SOEP data. In line with our results, the resilient type in Caliendo et. al. (2022a) also yields above-average values in all five traits. The under-controlled group, as in our study, has by far the lowest average scores in conscientiousness and agreeableness. Only in the case of the over-controlled group are there slight differences between our results and those of Caliendo et al. (2022a). For example, their over-controllers are characterized by relatively low extraversion scores, whereas in our study, the over-controllers’ extraversion score is close to the sample mean, which is likely a result of differences in how prototypes have been identified.^7^

**Table 1:** Big Five trait means by prototype (after LPA)

	Under-controllers	Over-controllers	Resilients	*Chi*^*2*^	
SOEP 2005					
Extraversion	− 0.48	− 0.03	0.80	2043.59	***
Conscientiousness	− 0.88	0.12	1.02	4444.83	***
Emotional stability	− 0.21	0.06	0.14	169.63	***
Openness to experience	− 0.04	− 0.08	0.43	417.41	***
Agreeableness	− 0.83	0.31	0.43	2177.60	***
*N*	3158	6163	2391		
SOEP 2009					
Extraversion	− 0.30	− 0.04	0.83	1415.32	***
Conscientiousness	− 0.85	0.19	1.12	4534.85	***
Emotional stability	− 0.08	0.01	0.14	60.69	***
Openness to experience	− 0.05	− 0.02	0.33	189.17	***
Agreeableness	− 0.79	0.32	0.60	2630.61	***
*N*	3563	5786	1765		
SOEP 2013					
Extraversion	− 0.71	− 0.03	0.93	1818.84	***
Conscientiousness	− 1.16	0.02	1.08	3312.10	***
Emotional stability	− 0.13	0.02	0.14	58.33	***
Openness to experience	− 0.24	− 0.02	0.30	202.31	***
Agreeableness	− 0.84	0.17	0.37	1141.89	***
*N*	1834	6182	1519		
SOEP 2017					
Extraversion	− 0.69	0.22	0.91	3789.9	***
Conscientiousness	− 0.70	0.18	1.16	5341.6	***
Emotional stability	− 0.18	0.03	0.19	261.3	***
Openness to experience	− 0.35	− 0.01	0.58	1018.0	***
Agreeableness	− 0.57	0.21	0.17	1849.0	***
*N*	5666	8545	1511		
SOEP 2019					
Extraversion	− 0.60	0.06	0.98	1401.74	***
Conscientiousness	− 0.78	0.21	1.20	2403.64	***
Emotional stability	− 0.36	0.10	0.08	257.47	***
Openness to experience	− 0.25	− 0.07	0.30	171.60	***
Agreeableness	− 0.75	0.23	0.62	1355.21	***
*N*	3994	9379	1493		

The table displays mean values for the prototypes (Big Five factor scores that are standardized to a mean of 0 and standard deviation of 1). The overall sample mean for each BF-Score is zero.

Statistical significance of cluster differences reported as ***significance level of 1%; **significance level of 5%

We also check if and how strongly the three personality types depend solely on stress resistance as one of the 15 Big Five items examined (BF 15 on the ability to cope with stress), as this could be considered a direct measure of resilience. Table 6 in the Appendix shows that stress resistance is lowest in undercontrollers and highest in individuals of the resilient prototype. This result is largely expected as resilient individuals generally display higher BF values than both under- and over-controllers. We take this as evidence of the principle content validity of the label ‘Resilient Prototype’. When we then regress the binary resilient personality variable against stress resistance, we further find that a one-standard deviation increase is related to 5% increase in the likelihood of being a member of the resilient prototype group. However, the R-squared value is only 2.7%. We therefore conclude that stress resistance represents only one component (among others) of what constitutes the resilient personality type.

#### Robustness test: results from the cluster analysis

In an analogous manner, Table 2 shows the findings of our cluster analysis in terms of the Big Five trait means. The picture gained by the LPA is confirmed in all of its essential points. The resilient type is marked by above-average values for each of the Big Five traits. Moreover, the resilient-type displays the highest probability of being self-employed. Nonetheless, there are also differences when compared to the LPA results. First, the relative size of the resilient group identified by means of cluster analysis (about 40% on average, see Table 7) is higher than that of the ‘LPA-resilients’ (15%, Table 7). Ward’s method tends to be biased towards equally sized groups (Hair et al., 1998), which explains the relatively high percentage share of the resilients in case of the cluster analysis. Whereas LPA follows a top-down-approach via a probabilistic model, examining which selection of groupings best fits the data, cluster analysis focuses on finding similarities/correlations between the observations from a bottom-up perspective. Second, the clustering results regarding the under- and over-controllers are somewhat different compared to the LPA. As expected, the group of under-controllers shows particularly low values regarding conscientiousness and agreeableness. However, depending on the year of the survey, the over-controllers have lower scores in terms of extraversion, openness or emotional stability.

**Table 2:** Big Five trait means by prototype (after cluster analysis)

	Under-controllers	Over-controllers	Resilients	Chi^2^	
SOEP 2005					
Extraversion	− 0.04	− 0.93	0.72	5114.53	***
Conscientiousness	− 0.91	0.30	0.49	3328.99	***
Emotional stability	− 0.21	− 0.13	0.24	407.85	***
Openness to experience	− 0.04	− 0.48	0.44	1533.39	***
Agreeableness	− 0.87	0.35	0.40	2853.42	***
*N*	3296	3470	4946		
SOEP 2009					
Extraversion	0.05	− 0.97	0.74	5336.34	***
Conscientiousness	− 0.95	0.22	0.49	3296.71	***
Emotional stability	− 0.06	− 0.18	0.18	226.41	***
Openness to experience	− 0.08	− 0.44	0.46	1490.60	***
Agreeableness	− 0.89	0.31	0.40	2896.80	***
*N*	3148	3438	4528		
SOEP 2013					
Extraversion	0.02	− 1.02	0.73	4402.10	***
Conscientiousness	− 1.08	0.22	0.48	3210.46	***
Emotional stability	− 0.23	− 0.14	0.28	460.09	***
Openness to experience	− 0.07	− 0.48	0.38	1066.22	***
Agreeableness	− 0.75	0.25	0.33	1799.14	***
*N*	2676	2867	3992		
SOEP 2017					
Extraversion	− 1.13	0.43	0.52	5886.47	***
Conscientiousness	− 0.41	− 0.12	0.34	1050.91	***
Emotional stability	− 0.05	− 0.91	0.71	5187.97	***
Openness to experience	− 0.55	0.32	0.01	1327.48	***
Agreeableness	− 0.17	− 0.26	0.16	409.94	***
*N*	4908	5025	5789		
SOEP 2019					
Extraversion	− 1.05	0.40	0.59	4116.48	***
Conscientiousness	− 0.27	0.01	0.38	525.47	***
Emotional stability	0.00	− 0.98	0.78	3965.36	***
Openness to experience	− 0.60	0.29	0.08	1042.99	***
Agreeableness	− 0.18	− 0.10	0.27	322.59	***
*N*	4611	4784	5471		

The table displays mean values for the three prototypes (Big Five factor scores that are standardized to a mean of 0 and standard deviation of 1). The overall sample mean for each BF-Score is zero

Statistical significance of cluster differences reported as ***significance level of 1%; **significance level of 5%

In summary, we find that the LPA and cluster analysis yield similar results. Most importantly, the results from both classification methods are particularly consistent when it comes to identifying the resilient type, which we expect to positively relate to entrepreneurial action. While our analysis clearly points toward a three-prototype solution — which is in line with previous research — it should be noted that two recent papers present a four- or five-prototype solution (Gerlach et al., 2018; Kerber et al., 2021). Nevertheless, these papers consistently identify a resilient type, as does our analysis. The variable on the resilient type therefore takes centre stage in the following regression analysis.

### Regression analysis

Prior to regressing variables pertaining to entrepreneurial action against personality prototypes, we follow Caliendo et al. (2014) by estimating the impact of the individual Big Five personality traits on self-employment decisions. The results — reported in Table 8 in the Appendix — are similar but not identical to their findings. According to Caliendo et al. (2014), there are two Big Five traits that increase self-employment (extraversion and openness). In the case of entry, the authors find positive and statistically significant effects of openness and extraversion. Our results fully support the former and partially support the latter. None of the Big Five traits are related to exit in our analysis, whereas Caliendo et al. (2014) find a positive effect of the agreeableness trait. We assume that differences in obtaining the Big Five traits are the main reason for the slight differences in regression results between our study and that of Caliendo et al. (2014).^8^

In a next step, we employ the LPA prototype variables in the regression analysis (see Table 3). Apart from specification 3 — which contains OLS results — all columns present average marginal effects after logit regressions. The LPA resilient type positively affects the probability of being self-employed by 1.6 percentage points, relative to the under-controlled type, which is omitted from the regression (Specification 1). Given the baseline probability of 8.5% in the sample (i.e. the mean Y-outcome in case of self-employed), the effect size must be deemed moderate to strong. Similarly, if an individual is classified as the LPA resilient type (as opposed to being under-controlled), the likelihood of entry increases by 0.2 percentage points (Specification 2). Again, given that the baseline probability is 0.8% in this case, the effect size can be considered quite strong. While the average number of entries is 0.063, being of the resilient type increases it by 0.011 (Specification 3). Furthermore, the LPA resilient type does not affect the likelihood of exit. Overall, the effect sizes are well within the range of what could have been expected based on the results of Caliendo et al. (2022a). By contrast, the over-controlled personality type exerts a negative effect on self-employment status and a positive effect on the number entries. Both effect sizes are quite small, however. It can therefore be stated that over- and under-controlled individuals display roughly similar propensities for self-employment.

**Table 3:** Regression results, LPA prototypes and entrepreneurship

	(1)	(2)	(3)	(4)
	Self-employed	Entry	Number of entries	Exit
Resilients	0.016*** (0.000)	0.002** (0.031)	0.011*** (0.000)	0.008 (0.368)
Over-controllers	− 0.004** (0.039)	− 0.000 (0.950)	0.003* (0.087)	0.007 (0.326)
LOC	0.017*** (0.000)	0.001** (0.019)	0.004*** (0.001)	− 0.012*** (0.001)
Risk tolerance	0.013*** (0.000)	0.002*** (0.000)	0.011*** (0.000)	0.001 (0.482)
Age *(not reported)*^*a*^				
Age squared *(not reported)*^*a*^				
University	0.025*** (0.000)	0.005*** (0.000)	0.019*** (0.000)	− 0.006 (0.453)
Vocational training	− 0.005** (0.016)	0.001 (0.150)	0.002 (0.420)	− 0.011 (0.170)
Full-time employment	0.042*** (0.000)	− 0.016*** (0.000)	− 0.026*** (0.000)	− 0.085*** (0.000)
Part-time employment	− 0.022*** (0.000)	− 0.012*** (0.000)	− 0.035*** (0.000)	− 0.042*** (0.000)
Female	− 0.014*** (0.000)	− 0.004*** (0.000)	− 0.008*** (0.000)	0.025*** (0.000)
Unemployed	− 0.066*** (0.000)	− 0.003*** (0.003)	− 0.023*** (0.000)	0.055*** (0.000)
Foreigner	− 0.001 (0.691)	0.001 (0.319)	0.016*** (0.000)	0.004 (0.705)
Experience work	− 0.001*** (0.000)	− 0.000 (0.865)	− 0.002*** (0.000)	− 0.001 (0.331)
Experience unemployed	− 0.001* (0.050)	− 0.000*** (0.002)	0.001*** (0.005)	0.004*** (0.006)
High school	0.037*** (0.000)	0.005*** (0.000)	0.037*** (0.000)	− 0.023*** (0.004)
Disability	− 0.001*** (0.000)	− 0.000** (0.021)	− 0.000*** (0.000)	0.001*** (0.006)
Father self-employed	0.039*** (0.000)	0.002* (0.089)	0.016*** (0.000)	− 0.017* (0.073)
North	− 0.007*** (0.003)	− 0.001 (0.210)	− 0.001 (0.553)	− 0.002 (0.803)
East	0.008*** (0.001)	0.000 (0.976)	0.009*** (0.000)	− 0.011 (0.160)
South	− 0.006*** (0.003)	− 0.001 (0.122)	0.007*** (0.000)	0.003 (0.689)
Capital income	0.069***	0.002***	0.019***	− 0.004
*(per increments of 100 k)*	(0.001)	(0.000)	(0.001)	(0.670)
*N*	111,559	95,071	111,559	8928
Mean Y-outcome	0.085	0.008	0.063	0.007

Robust standard errors, clustered on the individual level, are given in parentheses. Controls for each survey year are included. Specifications 1, 2, and 4 display marginal effects after logit regressions. Specification 3 displays OLS coefficients. In Table 12, results are displayed without control variables with the exception of the LOC/risk–variables.

*p*-values in parentheses: *p < 0.10; **p < 0.05; ***p < 0.01.

^a^A visualization of the non-linear relationship between age and the probability of different entrepreneurial decisions can be found in the Appendix (see Figure 6).

Table 9 in the Appendix displays the results of a similar regression analysis. This time, we use the prototype variables generated via clustering for the sake of testing the robustness of the LPA’s results. The average marginal effects are similar to those in the previous table and similarly support our main hypothesis. If an individual is of the resilient type, he or she is 1.6 percentage points more likely to be self-employed, 0.2 percentage points more likely to entry, and the number of total entries rises by 0.014. Again, we do not observe a relationship between the resilient type and the likelihood of exiting self-employment. In accordance with our results above, we find evidence of a negative relationship between over-controlled persons and self-employment (Table  9). The likelihood of self-employment is reduced by 0.5 percentage points when a person is over-controlled, which is quite similar to the regression results based on the LPA prototype variables (Table 3). In contrast to the LPA-based results above, the number of total entries falls by 0.004. However, we must not draw strong conclusions from this finding because its effect size is rather small. Nevertheless, it speaks to the fact that the over- and under-controller types are somewhat different depending on whether they are constructed via the LPA or Ward clustering/k-means procedure. We consequently focus our analysis on the resilient type as it can be clearly identified in both LPA and cluster analysis procedures. In addition, there is an on-going debate about the number of types, and the resilient type represents the only type that is repeatedly and consistently found by all previous research. Thus, based on the regression evidence, we conclude that the resilient prototype positively affects entrepreneurial activity.

In a next step, we employ both the entrepreneurial profile and the resilient prototype as explanatory variables in the regression analysis. Our findings suggest that the resilient type provides explanatory power beyond what can already be explained by the entrepreneurial profile (see Table 4). Regardless of whether we use LPA or cluster analysis, the resilient type remains statistically significant and positively associated with self-employment and entry when we control for the entrepreneurial profile. The effect sizes are thus well within the range of what is found in Caliendo et al. (2022a).

**Table 4:** Regression results, entrepreneurial profile and prototypes

	(1)	(2)	(3)	(4)	(5)	(6)	(7)	(8)
	Self-employed	Self-employed	Entry	Entry	Number of entries	Number of entries	Exit	Exit
Entrepreneurial profile *(per increments of 100)*	0.008*** (0.000)	0.007*** (0.000)	0.001*** (0.000)	0.001*** (0.000)	0.007*** (0.000)	0.005*** (0.000)	− 0.003 (0.282)	− 0.003 (0.216)
Resilients	0.015*** (0.000)	0.013*** (0.000)	0.002* (0.087)	0.002** (0.020)	0.004* (0.059)	0.010*** (0.000)	0.004 (0.572)	0.006 (0.374)
Prototype identification	LPA	Cluster analysis	LPA	Cluster analysis	LPA	Cluster analysis	LPA	Cluster analysis
*N*	111,559	111,559	95,071	96,029	111,559	111,559	8925	8925
Mean Y-outcome	0.085	0.085	0.008	0.008	0.063	0.063	0.007	0.007

Robust standard errors, clustered on the individual level, are given in parentheses. Controls for each survey year are included. Specifications 1, 2, 3, 4, 7 and 8 display marginal effects after logit regressions. Specifications 5 and 6 display OLS coefficients.

*p*-values in parentheses: *p < 0.10; **p < 0.05; ***p < 0.01.

One may object that the prototype variables and the entrepreneurial profile are not on the same measurement scale. The latter is a metric measure of the distance from a hypothetical situation in which the agreeableness trait takes the lowest possible value and the other four traits the highest possible values. On the other hand, the resilient prototype is a dummy variable that takes the value of one if the probability of belonging to that particular type is higher than the probability of belonging to either of the other two types. Therefore, to counter this potential objection and render effect sizes comparable, we equalize the measurement scale by generating the distance from a hypothetical resilient profile, which takes extreme positive values for all five traits. As a robustness check, we re-run the regression analysis using both this resilient profile and the entrepreneurial profile as our main explanatory variables (see Table 10 in the Appendix). We find that the smaller the distance to the hypothetical resilient reference profile, the higher the likelihood of self-employment and entry, as well as the number of entries. There is no longer a statistically significant relationship between the entrepreneurial profile and the likelihood of entry, although the probability of being self-employment and the number of entries are still positively associated with it. As before, there is no relationship between the resilient profile and the likelihood of exit. The results of this robustness test thus confirm that personality prototypes contribute to explaining entrepreneurial action beyond profiling.

## Conclusion

There is an established body of literature on the relationship between Big Five personality traits and self-employment. There is also highly relevant but hitherto underutilized literature from the field of psychology that moves from the trait-oriented approach to a person-oriented level of the Big Five, recognizing that there are stable and frequently occurring combinations of traits that exist in individuals in the general population (so-called personality prototypes). In this paper, we seek to bridge the gap between the prototyping and entrepreneurship literature by presenting evidence of a positive relationship between one particular prototype — i.e. the resilient type — and entrepreneurial activity.

The results of our empirical analysis reveal the existence of three Big Five prototypes in the German SOEP data. While there is an on-going debate about methods and the correct number of types, all previous research recognizes the resilient type (high levels in all five traits), which we also find, and which we argue to play a particularly important role in entrepreneurial activity. In fact, we find a positive and moderate to strong relationship between the resilient type and the likelihood of being self-employed, as well as the likelihood of entering into self-employment. Our results also show that the resilient prototype explains entrepreneurial activity over and above what can already be explained by the ‘entrepreneurial profile’. This finding implies that the profiling approach is only a first step on the way from the standard trait-level theorizing to a person-oriented perspective on the Big Five in the context of entrepreneurship. Hence, the present paper expands upon the profiling approach by suggesting that there are potentially several other combinations of the Big Five that may be connected to entrepreneurship.

**Fig. 5:**
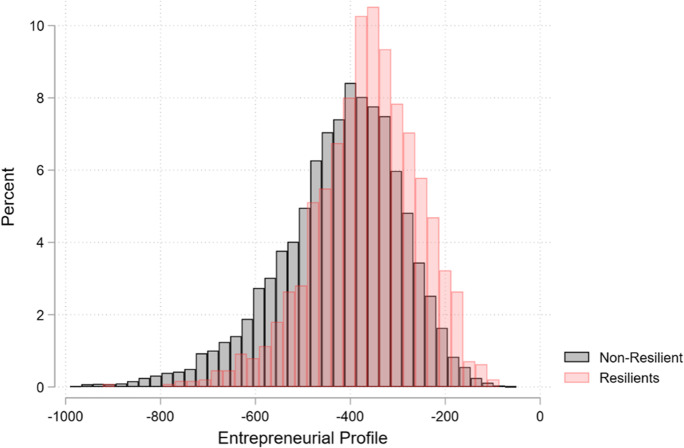
Share of incorrect predictions contingent on the number of prototypes. We perform a split-sample cross-validation procedure. First, the sample is randomly partitioned into two equally-sized subsamples, a subsample A (the calibration dataset) and B (the validation dataset). As a next step, an LPA is conducted based on subsample A and all model parameters are retained. Subsequently, we turn to subsample B, whereby first, the retained model parameters are used for predicting whether an individual belongs to a certain prototype (i.e. the constrained prediction). Second, the LPA is performed without fixing the parameters (i.e. the unconstrained prediction). Finally, we compare the constrained and unconstrained predictions. As the subsample selection is random, we repeat this process twenty times. The graph depicts the share of incorrect predictions depending on the number of prototypes.

**Fig. 6:**
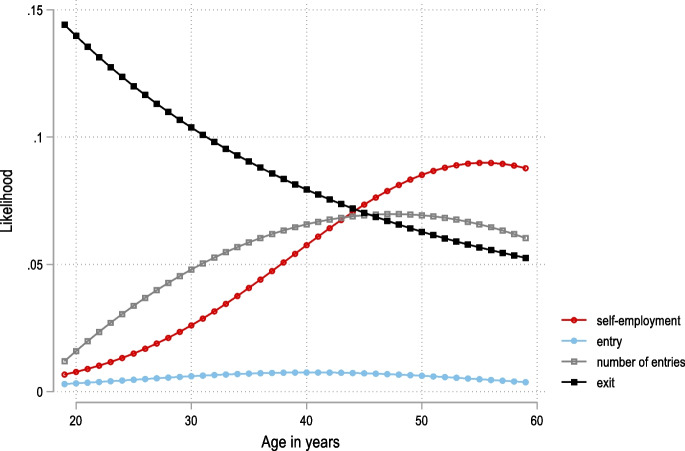
Marginal and non-linear effects of age (corresponds to Table 3)

Figure 5 displays a histogram of the distance to the hypothetical entrepreneurial profile for the resilient type and an aggregate of the other two prototypes. Members of the resilient type are on average closer to the entrepreneurial profile. Nevertheless, the most important finding here pertains to the fact that a large share of the resilient type members does not resemble the entrepreneurial profile at all. At the same time, the regression results above suggest that being of the resilient type makes it considerably more likely to engage in entrepreneurship. Thus, we conclude that the entrepreneurial profile ignores a large number of individuals who exhibit certain combinations of traits, some of which predispose them to become entrepreneurs. In addition, neither our LPA nor cluster analysis identifies the combination of traits labelled as the entrepreneurial profile, which indicates that it does not represent a combination of traits that frequently exists in the general population. In fact, there is not a single individual in our dataset who has an agreeableness score less than one standard deviation below the mean and more than one standard deviation above the mean for each other trait scores. By contrast, prototyping generates combinations of traits that are much less extreme than the hypothetical entrepreneurial profile and are thus more likely to describe actually existing personality patterns. In fact, the size of the group of individuals who fall into the resilient category is non-trivial according to our results, whereas the number of individuals who closely resemble the entrepreneurial profile is quite small.

In practice, this means that advice from career or business start-up advisors on personality should not be based on profiling alone. Otherwise, too many entrepreneurs might be discouraged from their entrepreneurial aspirations. In any case, it should be taken into account that the ‘right’ or ‘wrong’ personality is certainly not the one decisive factor for the start-up success of businesses, and that advice should never be given on the basis of personality alone. In this way, our paper complements the study of Konon and Kritikos (2019).

This leads to the need for further research. As the research on personality prototypes is an on-going process and statistical tools continue to be developed and refined, it is likely that additional combinations of stable and frequently occurring combinations of Big Five traits will emerge in the future. This prospect provides a promising avenue for future research efforts on the present topic. While the entrepreneurial profile has been repeatedly shown to display a positive association with entrepreneurship, our findings suggest that this approach is too narrow and represents only a first step. There is at least one other configuration of Big Five traits that displays a propensity for entrepreneurial action, and it is likely there are more that remain to be discovered. Finally, another interesting area of entrepreneurship research could only be briefly touched upon in this paper: The relationship between the prototype approach to personality and the psychological concept of resilience. Recently, a number of papers have been published on the resilience of entrepreneurs or small firms, e.g. in terms of business survival (Chadwick et al. 2020) or regarding the effects of the COVID-19 pandemic (Belitski et al., 2022; Caliendo et al., 2022b; Hadjielias et al., 2022). The present paper provides initial indications that persons of the resilient prototype have a higher psychological resilience in the narrow sense, which is why it is to be expected that they are better able to cope with crises than persons of the other two personality prototypes. However, further research is needed to substantiate this hypothesis.

## Figures and Tables

**Fig. 7:**
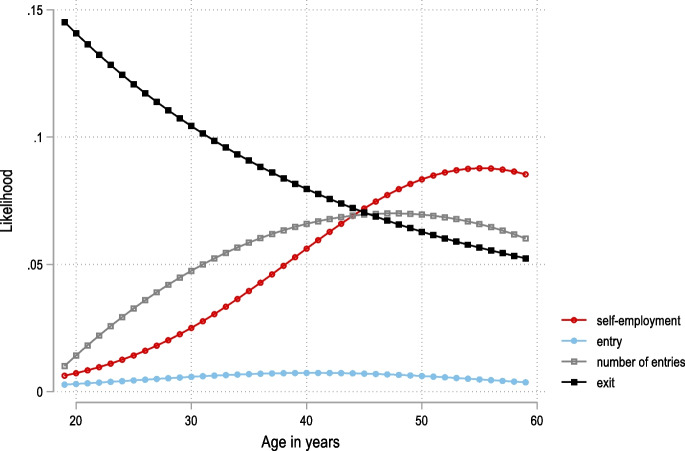
Marginal and non-linear effects of age (corresponds to Table 8)

## Data Availability

The Socioeconomic data is protected and cannot be freely shared but researchers can obtain it at the DIW Berlin. https://www.diw.de/en/diw_01.c.615551.en/research_infrastructure__socio-economic_panel__soep.html

## Appendix

Table 5

**Table 5:** Factor loadings after factor analysis (SOEP wave 2005)

	Extraversion	Conscientiousness	Neuroticism (emotional stability)	Openness	Agreeableness
Thorough	0.13	0.66	− 0.02	0.05	0.11
Communicative	0.66	0.21	− 0.04	0.14	0.09
Too rough	0.05	− 0.09	0.15	0.15	− 0.48
Inventive	0.37	0.20	− 0.08	0.50	− 0.08
Worried	− 0.02	0.11	0.50	0.05	0.09
Forgiving	0.16	0.15	− 0.01	0.10	0.39
Lazy	− 0.06	− 0.45	0.06	0.16	− 0.18
Social	0.67	0.10	− 0.06	0.21	0.11
Artistic	0.21	0.07	0.03	0.41	0.15
Nervous	− 0.06	− 0.07	0.63	0.04	− 0.04
Efficient	0.17	0.60	− 0.06	0.18	0.14
Reserved	− 0.48	0.08	0.15	0.05	0.22
Friendly	0.15	0.28	− 0.01	0.12	**0.5**8
Imaginative	0.32	0.04	0.01	0.52	0.09
Stress resilient	0.15	0.15	− 0.51	0.21	0.15

Source: SOEP, own calculation. Inverting the neuroticism value scale yields the variable emotional stability.

Table 6

**Table 6:** Mean values for Big Five item ‘stress resistance’

	LPA_under-controllers	LPA_over-controllers	LPA_resilients
2005	3.98	4.63	5.12
2009	4.10	4.53	5.06
2013	4.00	4.59	5.12
2017	4.05	4.77	5.35
2019	3.93	4.83	5.28

The survey question reads ‘I deal well with stress’ and ranges from 1 (not well at all) to 7 (very well).

Table 7

**Table 7:** Variable overview and descriptive statistics

Variable name	Description	Mean	Standard deviation
Self-employed	Dummy for being self-employed	0.085	0.279
Entry	Dummy for entry into self-employment	0.008	0.090
Number of entries	Number for entries into self-employment	0.063	0.274
Exit	Dummy for exit decision from self-employment	0.007	0.084
Extraversion	*Metric factor scores of the Big Five personality traits*	0.000	1
Conscientiousness		0.000	1
Emotional stability		0.000	1
Openness		0.000	1
Agreeableness		0.000	1
LPA_resilients	*Prototypes derived from Latent profile analysis. Type membership (exclusive) as binary variable*	0.154	0.361
LPA_over-controllers		0.567	0.496
LPA_under-controllers		0.290	0.454
Cluster_resilients	*Prototypes derived from cluster analysis. Type membership (exclusive) as binary variable*	0.403	0.490
Cluster_over-controllers		0.306	0.461
Cluster_under-controllers		0.291	0.454
Entrepreneurial profile	Distance to entrepreneurial profile	− 395.230	128.296
LOC	Locus of control (factor score after factor analysis)	0.017	0.816
Risk tolerance	On a Likert scale (0–10)	4.831	2.143
Age	In years	44.018	9.412
University	Dummy for having a university degree	0.240	0.427
Vocational training	Dummy for individuals who finished an apprenticeship	0.751	0.433
Full-time work	Dummy for full-time work	0.594	0.491
Part-time work	Dummy for part-time work	0.202	0.402
Female	Dummy for females	0.544	0.498
Unemployed	Dummy for individuals not in paid work	0.146	0.353
Foreigner	Dummy for non-German nationality	0.079	0.270
Experience work	Full-time work experience prior to the year of observation	15.090	10.776
Experience unemployed	Years of unemployment experience prior to the year of observation	1.214	2.829
High school	Dummy for individuals who received a diploma from a secondary school qualifying for university entrance	0.229	0.420
Disability	Degree of disability in per cent	3.106	12.960
Father self-employed	Dummy for having a father who was self-employed when the respondent was 15 years old	0.093	0.290
Capital income	Household income from asset flows, Euros per year	2265.428	18,308.130
North	Dummy for individuals living in the North of Germany	0.163	0.369
East	Dummy for individuals living in the East Germany	0.234	0.424
West	Dummy for individuals living in the West Germany	0.330	0.470
South	Dummy for individuals living in the South Germany	0.273	0.445

Source: The sample of the first model specification in Table 4 (dep. var. self-employed, *n* = 111,559) has been used to calculate the descriptive statistics. See Table 11 for a correlation matrix.

Table 8

**Table 8:** Regression results, Big Five factor scores and entrepreneurship

	(1)	(2)	(3)	(4)
	Self-employment	Entry	Number of Entries	Exit
Extraversion	0.011*** (0.000)	0.000 (0.242)	0.005*** (0.000)	− 0.001
				(0.829)
Conscientiousness	− 0.002* (0.093)	0.000 (0.208)	− 0.003*** (0.003)	0.000 (0.964)
Emotional stability	− 0.004*** (0.000)	0.000 (0.918)	0.001 (0.538)	− 0.000 (0.945)
Openness	0.015*** (0.000)	0.002*** (0.000)	0.015*** (0.000)	− 0.003 (0.340)
Agreeableness	0.000 (0.715)	0.000 (0.912)	0.001 (0.300)	0.003 (0.386)
LOC	0.018*** (0.000)	0.001** (0.032)	0.004*** (0.001)	− 0.012*** (0.003)
Risk tolerance	0.011*** (0.000)	0.001*** (0.000)	0.009*** (0.000)	0.002 (0.321)
*Age (not reported)*^*a*^				
*Age squared (not reported)*^*a*^				
University degree	0.023*** (0.000)	0.004*** (0.000)	0.016*** (0.000)	− 0.006 (0.433)
Vocational training degree	− 0.006*** (0.004)	0.001 (0.161)	0.001 (0.640)	− 0.011 (0.171)
Full-time employment	0.042*** (0.000)	− 0.016*** (0.000)	− 0.026*** (0.000)	− 0.085*** (0.000)
Part-time employment	− 0.021*** (0.000)	− 0.012*** (0.000)	− 0.034*** (0.000)	− 0.042*** (0.000)
Female	− 0.019*** (0.000)	− 0.004*** (0.000)	− 0.010*** (0.000)	0.026*** (0.000)
Unemployed	− 0.066*** (0.000)	− 0.003*** (0.002)	− 0.023*** (0.000)	0.055*** (0.000)
Foreigner	− 0.003 (0.471)	0.001 (0.347)	0.016*** (0.000)	0.005 (0.676)
Experience work	− 0.001*** (0.000)	− 0.000 (0.894)	− 0.002*** (0.000)	− 0.000 (0.352)
Experience unemployed	− 0.001 (0.168)	− 0.000*** (0.004)	0.001*** (0.001)	0.004*** (0.006)
High school	0.035*** (0.000)	0.005*** (0.000)	0.035*** (0.000)	− 0.022*** (0.005)
Disability	− 0.001*** (0.000)	− 0.000** (0.026)	− 0.000*** (0.001)	0.001*** (0.007)
Father self-employed	0.038*** (0.000)	0.002* (0.072)	0.016*** (0.000)	− 0.017* (0.077)
North	− 0.004 (0.169)	− 0.000 (0.876)	− 0.003 (0.249)	− 0.005 (0.622)
East	0.012*** (0.000)	0.001 (0.179)	0.009*** (0.000)	− 0.014 (0.101)
South	0.004** (0.034)	0.001 (0.172)	0.007*** (0.001)	− 0.003 (0.680)
Capital income	0.069*** (0.000)	0.002*** (0.000)	0.019*** (0.001)	− 0.005 (0.638)
*N*	111,559	95,082	111,559	8928
Mean Y-outcome	0.085	0.008	0.063	0.007

Robust standard errors, clustered on the individual level, are given in parentheses. Controls for each survey year are included. Specifications 1, 2, and 4 display marginal effects after logit regressions. Specification 3 displays OLS coefficients.

*p*-values in parentheses: **p* < 0.10; ***p* < 0.05; ****p* < 0.01.

^a^A visualization of the non-linear relationship between age and the probability of different entrepreneurial decisions can be found in the Appendix (see Figure 7).

Table 9

**Table 9:** Regression results, cluster analysis’ prototypes and entrepreneurship

	(1)	(2)	(3)	(4)
	Self-employed	Entry	Number of entries	Exit
Resilients	0.016***	0.002**	0.014***	0.001
	(0.000)	(0.017)	(0.000)	(0.865)
Over-controllers	− 0.005**	− 0.000	− 0.004**	− 0.004
	(0.026)	(0.937)	(0.026)	(0.619)
*N*	111,559	95,071	111,559	8928
Mean Y-outcome	0.085	0.008	0.063	0.007

Robust standard errors, clustered on the individual level, are given in parentheses. Controls for each survey year and the full set of covariates are included. Specifications 1, 2, and 4 display marginal effects after logit regressions. Specification 3 displays OLS coefficients.

*p*-values in parentheses: **p* < 0.10; ***p* < 0.05; ****p* < 0.01.

Table 10

**Table 10:** Regression results, entrepreneurial profile and resilient profile

	(1)	(2)	(3)	(4)
	Self-employed	Entry	Number of entries	Exit
Resilient profile	0.006***	0.001***	0.006***	0.002
	(0.000)	(0.004)	(0.000)	(0.629)
Entrepreneurial profile	0.005***	0.000	0.003***	-0.004
	(0.000)	(0.200)	(0.000)	(0.264)
*N*	111,559	95,071	111,559	8928
Mean Y-outcome	0.085	0.008	0.063	0.007

Robust standard errors, clustered on the individual level, are given in parentheses. Controls for each survey year and the full set of covariates are included. Specifications 1, 2, and 4 display marginal effects after logit regressions. Specification 3 displays OLS coefficients.

*p*-values in parentheses: * *p* < 0.10; ** *p* < 0.05; *** *p* < 0.01.

Table 11

**Table 11:** Correlation matrix (self-employed sample)

		1	2	3	4	5	6	7	8	9	10	11	12	13	14	15	16	17	18	19	20	21	22
1	Self-employed	1.00																					
2	Number of entries	0.40	1.00																				
3	Resilients	0.03	0.01	1.00																			
4	Over-controllers	− 0.02	0.00	− 0.49	1.00																		
5	Under-controllers	0.00	− 0.01	− 0.27	− 0.71	1.00																	
6	Entrepr-Profile	0.09	0.06	0.14	0.00	− 0.11	1.00																
7	Resilient Profile	0.08	0.05	0.32	0.22	− 0.51	0.68	1.00															
8	LOC	0.09	0.03	0.08	0.06	− 0.12	0.21	0.29	1.00														
9	Risk	0.11	0.08	0.07	− 0.02	− 0.03	0.27	0.22	0.10	1.00													
10	Age	0.07	0.01	0.03	0.01	− 0.03	0.00	0.01	− 0.04	− 0.07	1.00												
11	University	0.13	0.07	− 0.06	0.03	0.01	0.08	0.08	0.13	0.02	0.06	1.00											
12	Vocationaö	− 0.06	− 0.03	0.04	0.00	− 0.04	0.02	0.00	0.00	− 0.02	0.09	− 0.43	1.00										
13	Full_time	0.13	0.00	0.00	− 0.02	0.02	0.14	0.07	0.12	0.13	0.01	0.12	0.04	1.00									
14	Part_time	− 0.07	− 0.01	0.01	0.04	− 0.05	− 0.05	0.01	0.00	− 0.10	0.05	− 0.02	0.03	− 0.61	1.00								
15	Female	− 0.09	− 0.01	0.06	0.06	− 0.11	− 0.10	0.03	− 0.03	− 0.19	− 0.01	− 0.04	− 0.01	− 0.49	0.37	1.00							
16	Unemployed	− 0.09	− 0.01	− 0.01	− 0.02	0.03	− 0.11	− 0.08	− 0.14	− 0.04	− 0.07	− 0.10	− 0.07	− 0.42	− 0.11	0.17	1.00						
17	Foreigner	− 0.02	0.00	0.00	0.00	− 0.01	− 0.02	0.00	− 0.08	0.01	− 0.06	− 0.02	− 0.20	− 0.06	− 0.02	0.01	0.10	1.00					
18	Experience Work	0.09	− 0.03	0.03	− 0.02	0.00	0.09	0.02	0.03	0.05	0.63	− 0.02	0.18	0.49	− 0.29	− 0.40	− 0.26	− 0.07	1.00				
19	Experience Unemployment	− 0.05	0.01	0.00	− 0.03	0.03	− 0.10	− 0.09	− 0.19	0.01	0.07	− 0.13	− 0.01	− 0.25	− 0.04	0.03	0.35	0.03	− 0.13	1.00			
20	High School	0.13	0.08	− 0.07	0.02	0.04	0.06	0.05	0.14	− 0.01	− 0.03	0.55	− 0.35	0.07	0.00	− 0.01	− 0.09	− 0.11	− 0.10	− 0.14	1.00		
21	Disability	− 0.04	− 0.02	0.01	− 0.03	0.03	− 0.07	− 0.08	− 0.09	− 0.03	0.12	− 0.05	0.01	− 0.05	− 0.02	− 0.03	0.05	− 0.02	0.08	0.08	− 0.06	1.00	
22	Father Self-employed	0.03	0.02	− 0.02	− 0.01	0.02	0.01	− 0.01	0.00	0.02	− 0.20	0.02	− 0.07	0.01	− 0.02	− 0.01	0.01	− 0.01	− 0.13	− 0.05	0.02	− 0.02	1.00
23	Capital income	0.09	0.02	− 0.01	− 0.01	0.02	0.01	0.01	0.04	0.02	0.05	0.06	− 0.02	0.01	0.00	− 0.01	− 0.01	− 0.01	0.02	− 0.04	0.06	0.00	0.03

Table 12

**Table 12:** Regression results, LPA prototypes and entrepreneurship (specifications without further control variables)

	(1)	(2)	(3)	(4)	(5)	(6)	(7)	(8)
	Self-employed	Self-employed	Entry	Entry	Number of entries	Number of entries	Exit	Exit
Resilient	0.022***	0.006**	0.002**	0.001*	0.008***	0.007**	− 0.000	0.008
	(0.000)	(0.026)	(0.017)	(0.097)	(0.000)	(0.016)	(0.985)	(0.381)
Over-controlled	− 0.001	− 0.009***	− 0.001	− 0.000	0.002	0.002	− 0.004	0.003
	(0.650)	(0.000)	(0.343)	(0.589)	(0.179)	(0.276)	(0.425)	(0.666)
LOC		0.028***		0.001***		0.006***		− 0.022***
		(0.000)		(0.005)		(0.000)		(0.000)
Risk		0.013***		0.001***		0.009***		− 0.002*
		(0.000)		(0.000)		(0.000)		(0.067)
*N*	145,662	105,319	132,899	96,435	145,662	105,319	12,657	8830
Mean Y-outcome	0.085	0.085	0.008	0.008	0.063	0.063	0.007	0.007

Robust standard errors, clustered on the individual level, are given in parentheses.

*p*-values in parentheses: **p* < 0.10; ***p* < 0.05; ****p* < 0.01.

## Footnotes

1 To prevent possible misunderstandings, it should be noted that the label ‘over-controlled’ must not be confused with, and is different from an individual’s desire for independence and personal control, which is a key motivation to enter self-employment.

2 ‘Socio-Economic Panel (SOEP), data for years 1984–2019, SOEP-Core v36, EU Edition, 2021, 10.5684/soep.core.v36eu.’.

3 For general information on the SOEP, see Giesselmann et al. (2019); Goebel et al. (2019); Schröder et al. (2020).

4 A number of studies have shown that Big Five personalities are relatively constant and stable over time (e.g. Specht et al., 2011; Lucas and Donnellan 2011; Cobb-Clark and Schurer 2011; Specht et al., 2014).

5 Factor loadings for the other years are not reported but follow the same pattern. The corresponding results are available upon request.

6 From 30 separate logit regressions, each containing two Big Five trait variable and their interaction term (10 possible combinations of the Big Five variables) and no further controls, performed separately for the three dependent variables (self-employment, entry and exit), the predicted probabilities of the three dependent variables are calculated for each pairwise interaction between two traits.

7 Caliendo et al. (2022a) do not measure the Big Five traits by using factor analysis but by averaging survey items, they use single linkage hierarchical clustering (instead of Ward’s linkages) followed by k-means clustering to measure prototypes, and they do not identify prototype solutions for individual survey years of the SOEP but for the complete sample instead.

8 Caliendo et al. (2014) assign the individual survey items directly to the expected traits, before obtaining ‘a respondent’s score for a trait or personality characteristic by averaging the scores from the different statements referring to that construct’ (p. 795). In contrast, we do not make such an a priori assignment. Instead, latent variables or factors are determined through factor analysis. As a result, the Big Five traits are extracted from the fifteen survey items by generating metric factor scores for each trait, which are weighted linear combinations of the 15 survey items.
